# Epidemiology of onchocerciasis-associated epilepsy in the Mbam and Sanaga river valleys of Cameroon: impact of more than 13 years of ivermectin

**DOI:** 10.1186/s40249-018-0497-1

**Published:** 2018-12-03

**Authors:** Joseph Nelson Siewe Fodjo, Godwin Tatah, Earnest Njih Tabah, Leonard Ngarka, Leonard Njamnshi Nfor, Samuel Eric Chokote, Michel K. Mengnjo, Fidèle Dema, Aurélien Tele Sitouok, Grace Nkoro, Félicien E. Ntone, Anne-Cécile Zoung-Kanyi Bissek, Cédric B. Chesnais, Michel Boussinesq, Robert Colebunders, Alfred K. Njamnshi

**Affiliations:** 1 Global Health Institute, University of Antwerp, Antwerp, Belgium; 2 Neurology Department, Yaoundé Central Hospital, Yaoundé, Cameroon; 3 Neurology Department, CH Saint-Nazaire, Saint-Nazaire, France; 4 Neuroscience Laboratory, FMBS, The University of Yaoundé I, Yaoundé, Cameroon; 5 Ministry of Public Health, Yaoundé, Cameroon; 6 Neurology Department, CHU Brugman, Brussels, Belgium; 7 Yoko District Hospital, Yaoundé, Cameroon; 8 UMI 233, Institut de Recherche pour le Développement (IRD), Montpellier, France; 9 Université Montpellier, Montpellier, France; 10 INSERM Unité 1175, Montpellier, France; 11 Brain Research Africa Initiative (BRAIN), Yaoundé, Cameroon

**Keywords:** Onchocerciasis, Epilepsy, Nodding syndrome, Ov16 rapid diagnostic test, Ivermectin, Cameroon

## Abstract

**Background:**

**A** high epilepsy prevalence has been reported in several onchocerciasis-endemic villages along the Mbam and Sanaga river valleys in Cameroon, including Bilomo and Kelleng. We sought to determine the prevalence of epilepsy in these two villages following more than 13 years of community-directed treatment with ivermectin (CDTI).

**Methods:**

Door-to-door surveys were performed on the entire resident population in the villages in August 2017 and January 2018. Epilepsy was diagnosed using a 2-step approach: administration of a standardized 5-item questionnaire followed by confirmation by a neurologist. Previously published diagnostic criteria for onchocerciasis-associated epilepsy (OAE) were used. Ov16 serology was done for children aged 7–10 years to assess onchocerciasis transmission. Findings were compared with previous data from these two villages.

**Results:**

A total of 1525 individuals (1321 in Bilomo and 204 in Kelleng) in 233 households were surveyed in both villages**.** The crude prevalence of epilepsy was 4.6% in Bilomo (2017) and 7.8% in Kelleng (2018), including 12 (15.6% of cases) persons with epilepsy (PWE) with nodding seizures. The age and sex-standardized prevalence in Kelleng decreased from 13.5% in 2004 to 9.3% in 2018 (*P* < 0.001). The median age of PWE shifted from 17 (IQR: 12–22) years to 24 (IQR: 20–30) years in Bilomo (*P* < 0.001); and slightly from 24 (IQR: 14–34) years to 28 (IQR: 21.25–36.75) years in Kelleng (*P* = 0.112). Furthermore, 47.6% of all tested children between 7 and 10 years had Ov16 antibodies.

**Conclusions:**

There is a decrease in epilepsy prevalence after 13 years and more of CDTI in both villages. The age-shift observed in PWE suggests that ivermectin may prevent OAE in younger residents. Ov16 seropositivity in children indicates ongoing onchocerciasis transmission possibly due to suboptimal control measures. Our findings support the existence of OAE in Cameroon and highlight the need to strengthen onchocerciasis elimination programs.

**Electronic supplementary material:**

The online version of this article (10.1186/s40249-018-0497-1) contains supplementary material, which is available to authorized users.

## Multilingual abstracts

Please see Additional file 1 for translations of the abstract into the five official working languages of the United Nations.

## Introduction

Currently, about 50 million people worldwide have epilepsy and nearly 80% of the persons with epilepsy (PWE) live in low and middle-income countries [1]. Studies in Cameroon have revealed an epilepsy prevalence ranging from 1.16 to 13.4% [2]. Onchocerciasis-endemic foci are of particular interest as previous studies reported an association between epilepsy and infection with *Onchocerca volvulus* [3–5]*.* Past surveys in two onchocerciasis-endemic villages in Cameroon reported a high epilepsy prevalence in Bilomo in 1998 [6] and in Kelleng in 2004 [7]. Recently, the term onchocerciasis-associated epilepsy (OAE) has been used to describe different forms of epilepsy including nodding seizures and “Nakalanga” features (various degrees of stunted growth, dysmorphic features, mental retardation and seizure disorders) that occur in areas of high onchocerciasis transmission [8, 9]. Cases of nodding seizures have been reported in Cameroon as well [7].

The mechanism by which *O. volvulus* may trigger epilepsy remains unclear; however strong epidemiological evidence suggests a possible causative role [4, 5]. Moreover, observations in the Democratic Republic of Congo (DRC) and northern Uganda suggest that onchocerciasis control through mass drug administration (MDA) of ivermectin may decrease the incidence of OAE [10, 11]. In Cameroon, annual community-directed treatment with ivermectin (CDTI) was instituted in the 1990s by the Ministry of Public Health [12]. In the present study we evaluated the epilepsy prevalence in two villages where a census of PWE had been conducted in the late 1990s/early 2000s and which had benefitted from more than a decade of ivermectin distribution, and we compared the results with those obtained during the initial surveys. In addition, we evaluated the impact of the ivermectin MDA on the transmission of onchocerciasis, using an antibody-detection rapid diagnostic test.

## Methods

### Study sites

**Fig. 1:**
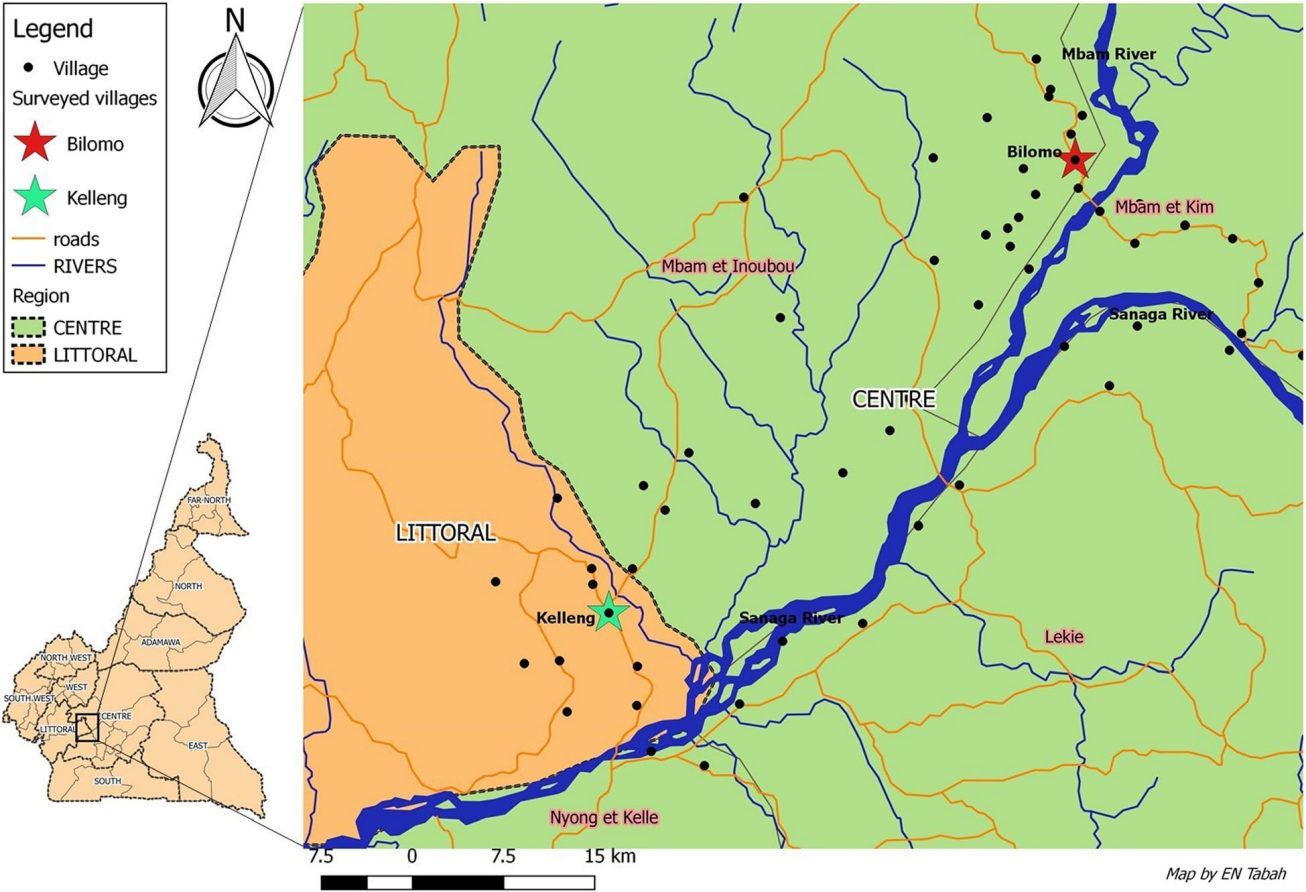
Map showing the locations of Bilomo and Kelleng

The study sites for the door-to-door surveys are two villages located along river banks in two different regions of Cameroon: Bilomo in the Centre region in the Mbam valley and Kelleng in the Littoral region in the Sanaga valley (Fig. 1).

Bilomo village is located 120 km north of Yaoundé, in the Centre Region of Cameroon. It is less than 2 km away from the banks of the Mbam river, a tributary of the Sanaga river with known breeding sites for blackflies, vectors of *O. volvulus*. Bilomo is located close to villages with confirmed very high levels of endemicity for onchocerciasis prior to the initiation of CDTI [3]. Mass treatment with ivermectin is done annually by the Ministry of Public Health since 1998 [12]. Most inhabitants practise subsistence farming and spend the most of their time in the fields, exposing themselves to blackfly bites. The closest health facility is 1 km away from the village and is run by a nurse, assisted by two community volunteers.

Kelleng is located about 100 km North-West of Yaoundé, in the Littoral Region of Cameroon. The huge Kikot rapids of the Sanaga River are located less than 5 km away from the village settlement and provide suitable breeding sites for blackflies; indeed, earlier surveys confirmed Kelleng as a hyperendemic focus for onchocerciasis [7]. Ivermectin was sporadically distributed by private parties in the early 1990s, but the government instituted the yearly distribution of ivermectin in the mid-1990s [12]. As in Bilomo, farming is the main activity of the inhabitants who have access to a health center with one nurse and an assistant.

### Study procedures

#### Baseline surveys

From August to mid-October 1998, a cross-sectional survey was carried out by a research team from the Faculty of Medicine and Biomedical Sciences of the University of Yaoundé I with the aim of determining the prevalence, associated factors and management of epilepsy in Bilomo village [13]. This was a door-to-door survey where suspected cases of epilepsy were identified by trained non-physician survey staff, using the questionnaire developed by the Institute of Neurological Epidemiology and Tropical Neurology of Limoges (France) and the Pan African Association of Neurological Sciences (PAANS) as screening instrument. This was followed by a confirmation of the epilepsy cases by neurologists (AKN et al.) through a complete clinical examination. Clinical evaluation for onchocerciasis was done by assessing the presence of nodules and onchodermatitis in PWEs. The crude epilepsy prevalence reported in this survey was 4.9% (93/1898) [6] and was one of the highest in Africa at that time, equal only to that of a study in Liberia [14]. Data on the age/sex structure of the Bilomo population in 1998 and the duration of epilepsy at the time of the survey was not collected. The results of this study were presented to the Ministry of Public Health of Cameroon by the authors and it led to the establishment of a national epilepsy control programme with epilepsy clinics in the Mbam area in the year 2000 [15].

Between July and August 2004, a door-to-door survey was carried out in Kelleng by a team of researchers mostly from the University of Rome La Sapienza, Italy [7]. The procedures were similar to those in Bilomo with a screening phase, a confirmation and a paraclinical phase. The screening was done by the team members (physicians) and confirmation of epilepsy cases by four physicians trained in child neurology. The screening instrument was an adaptation of 10 questions based on the questionnaire developed by the Institute of Neurological Epidemiology and Tropical Neurology of Limoges (France). A clinical evaluation of onchocerciasis was also done in PWE (presence of nodules, skin depigmentation). In this survey, only 181 of the 197 inhabitants were examined because 16 individuals were not available for interview. The age/sex structure of the entire village population was well reported, as well as the duration of epilepsy at the time of the survey from which the initial yearly incidence could be deduced. The crude prevalence rate of active epilepsy found in this study was 10.5% (19/181) and the age-adjusted (with respect to the 2000 Cameroon population) prevalence rate was 13.5%. However, the results of this study were never made available to the health authorities of the country nor to the national scientists until their publication in 2008 [7].

#### Current surveys

In August 2017 and January 2018, cross-sectional studies using a door-to-door approach were repeated in Bilomo and Kelleng villages respectively, by a team consisting of neurologists (AKN, TGY, LN, CSE), medical doctors with specialised epilepsy training (MMK, DF, SFJN, TSA), dermatologists (ACZKB, GN), a psychiatrist (FEN) and a neuro-epidemiologist (ENT). The survey lasted for 8 days in Bilomo and 2 days in Kelleng. In each village, we met the local authorities (village chief and nurse) who then linked us to community health workers and/or ivermectin distributors. The latter have a mastery of the village and served as our guides to the various households and interpreters when necessary. After obtaining signed informed consent from the head of the household or his/her representative, data was obtained from the participants as per the research instrument.

The research instrument was a 2-part family survey questionnaire; the first part for collecting demographic information about the family, duration of their stay in the village, deaths of PWE in the household and details about ivermectin treatment. We included only residents who had been living in the village for ≥6 months. The second part was a tool to screen for epilepsy in the household, using the 5-item questionnaire developed by the Institute of Neurological Epidemiology and Tropical Neurology of Limoges (France) for routine diagnosis [16]. This tool was designed for Africa with the support of the PAANS and the International League Against Epilepsy and was validated by Diagana et al. in Mauritania [16]. The five screening questions were: 1) history of loss of consciousness with or without loss of bladder control and/or drooling; 2) history of absence or sudden lapse of consciousness of brief duration; 3) history of sudden involuntary jerks of the arms and/or legs (convulsions) lasting for a few minutes; 4) history of sudden and brief bodily sensations, seeing or hearing things that are not there, or smelling strange odours; and 5) having been told that he/she had epilepsy or had experienced two epileptic episodes. Suspected cases of epilepsy screened by the general physicians were further evaluated by a neurologist or a physician with specialised training in epilepsy for confirmation. The specialised physicians revisited households with suspected cases of epilepsy who were absent during their first visit one or more times until they were able to meet and examine all the suspected cases.

Ongoing onchocerciasis transmission in the villages was assessed in children aged 7–10 years, using the SD Bioline Onchocerciasis Ov16 IgG4 rapid diagnostic test (RDT) (Standard Diagnostics, Gyeonggi-do, South Korea) for anti-*Onchocerca* antibodies [17].

### Definitions

Confirmed epilepsy was defined as the occurrence of at least two unprovoked seizures separated by 24 h or more, as adapted from the operational definition of the International League Against Epilepsy (ILAE) guidelines of 2014 [18]. This definition excludes any seizure related to an acute cause such as fever, acute metabolic imbalance, alcohol and substance abuse/withdrawal.

OAE was defined based on the diagnostic criteria published earlier [9], which include: 1) Person living for at least 3 years in an *O. volvulus*-endemic region; 2) High prevalence of epilepsy in the village and families having more than one child with epilepsy; 3) History of at least two unprovoked epileptic seizures 24 h apart; 4) No other obvious cause for the epilepsy (mainly established by good history taking to exclude common causes such as perinatal, infectious or traumatic); 5) Onset of epilepsy between the ages of 3 and 18 years; 6) Normal psycho-motor development before the onset of epilepsy. In case the person has never taken ivermectin, one of the following additional criteria may be useful: seropositivity for Ov16 antibodies or presence of microfilariae or DNA of *O. volvulus* in skin biopsies, or presence of onchocerciasis clinical manifestations (characteristic skin lesions and/or subcutaneous nodules).

A “native household” was defined as one in which the family head was born in the village and raised his family there. “Immigrant households” referred to those whose family heads were not born in the village, but came and settled there.

CDTI coverage was calculated as a percentage of the eligible population (aged ≥5 years and non-pregnant) that reported having taken ivermectin [19] in 2017.

### Ethical considerations

Prior to the study, ethical clearance was obtained from the University of Antwerp in Belgium (Registration number B300201731362) and the Cameroon National Ethics Committee for Research in Human Health (Registration number 2017/02/875/CE/CNERSH/SP). Administrative authorization was granted by the Ministry of Public Health of Cameroon (D30–177/L/MINSANTE/SG/DROS/TMC). The collaboration of local administrative and religious authorities was obtained for the research project. All participants signed an informed consent form and the data obtained was treated confidentially.

### Data analysis

Proportions were calculated for categorical variables and compared using Chi-squared tests. For continuous variables, median and interquartile ranges (IQR) or means and standard deviations were used as appropriate and compared using Wilcoxon-Mann-Whitney or Independent *t*-tests. The level of significance was set at *P* < 0.05, and all *P*-values were two-sided. Analyses were performed using SPSS version 20 (IBM, New York, USA), and WinPepi version 11.65 (PEPI Suite).

Crude incidence rates were calculated by considering in each village the number of persons with a duration of epilepsy between 0 and 5 years at the time of the survey and dividing by five to obtain yearly values. Previous incidence in Kelleng was estimated by considering all cases with duration of epilepsy between 0 and 5 years as published during the previous survey in 2004, and dividing by five; such data was not available for Bilomo in 1998. The epilepsy incidence rates were expressed per 100 000 person-years (PY).

### Standardization method

To ease comparison with previous studies, the standardized epilepsy prevalence in Kelleng was calculated using the 2010 national population age/sex structure (age groups: 0–9 years, 10–19 years, 20–29 years, 30–39 years, 40–49 years, ≥50 years), obtained using projections from the 3rd population and housing census of Cameroon [20]. A direct age/sex standardization was performed by applying the observed crude age-specific prevalence by sex rates for Kelleng in 2018, to the corresponding age/sex groups of the total 2010 Cameroon population [20], to get the expected number of epilepsy cases for each age/sex group. The sum of the expected number of epilepsy cases for each age/sex group was then divided by the total Cameroon population to obtain the standardized prevalence rates (Additional file 2). Prevalence rates were compared using the Chi-squared test.

Similarly, the age/sex population structure for Bilomo in 1998 was deduced by applying the demographic features observed during the 2017 survey in Bilomo, to the total population of Bilomo in 1998 as documented by the previous survey [6]. In both cases, we assumed a non-significant change in the population structures of both villages over the years.

## Results

### Study population

A total of 1525 individuals (1321 in Bilomo and 204 in Kelleng) were screened for epilepsy from 233 households (193 in Bilomo and 40 in Kelleng). All households of Kelleng participated in the survey; in Bilomo, two households (0.9%) were not included in the study because the occupants permanently resided in the city and spent only a few weeks in the study site every year. Table 1 gives detailed characteristics of the individuals and households surveyed in 2017/2018. Seven hundred and seventy-seven (51%) of the study population were females. The study population had a median age of 18 years (interquartile range (IQR): 8–37 years), with 65% below 30 years of age. The median household size was six persons with seven in Bilomo and five in Kelleng. The proportion of immigrant households was 11%, and their median length of stay in the villages was 11 years (IQR: 6–27). Agriculture constituted the main (90%) activity and source of household income. Forty-four (18.9%) of the 233 households had already lost a family member to epilepsy at a median age of 22 years (IQR: 18–25).

**Table 1:** Characteristics of households and individuals surveyed in 2017–2018

	Bilomo	Kelleng	Overall
Households	*n* = 193	*n* = 40	*N* = 233
Native households: *n* (%)	171 (89%)	36 (90%)	207 (89%)
Immigrant households: *n* (%)	22 (11%)	4 (10%)	26 (11%)
LOS** of immigrant households: median (IQR)	11 (6.25–28.5)	11 (7–12)	11 (6–27)
Median household size	7	5	6
Agriculture as main activity: *n* (%)	177 (92%)	33 (83%)	210 (90%)
Family history of death from epilepsy: *n* (%)	39 (20%)	5 (13%)	44 (19%)
Age at death of PWE: median (IQR)	22 (18–25)	20 (18-–25.25)	22 (18–25)
Participants	*n* = 1321	*n* = 204	*N* = 1525
Age: median (IQR)	18 (8–36)	21 (8.25–45)	18 (8–37)
Age distribution: n (%)			
0–9 years	409 (31%)	56 (27%)	465 (30%)
10–19 years	297 (22%)	43 (21%)	340 (22%)
20–29 years	170 (13%)	23 (11%)	193 (13%)
30–39 years	160 (12%)	24 (12%)	184 (12%)
40–49 years	85 (6%)	17 (8%)	102 (7%)
≥50 years	200 (15%)	41 (20%)	241 (16%)
Gender: *n* (%)			
Female	679 (51%)	98 (48%)	777 (51%)
Male	642 (49%)	106 (52%)	748 (49%)
Epilepsy cases			
Total number of confirmed PWE	*n* = 61	*n* = 16	*N* = 77
Crude epilepsy prevalence (95% *CI*)	4.6% (3.6–5.9)	7.8% (4.9–12.4)	5.1% (4.1–6.3)
Age and sex standardized prevalence	5.4%	9.3%	5.8%
Gender-specific prevalence: *n* (%)			
Female	29 (4.3%)	8 (8.2%)	37 (4.8%)
Male	32 (5.0%)	8 (7.5%)	40 (5.3%)
Age-specific prevalence: *n* (prevalence)			
0–9 years	1 (0.2%)	0 (0.0%)	1 (0.2%)
10–19 years	12 (4.0%)	2 (4.7%)	14 (4.1%)
20–29 years	29 (17.1%)	7 (30.4%)	36 (18.7%)
30–39 years	15 (9.4%)	4 (16.7%)	19 (10.3%)
40–49 years	3 (3.5%)	2 (11.8%)	5 (4.9%)
≥ 50 years	1 (0.5%)	1 (2.4%)	2 (0.8%)
Age in years, PWE only: median (IQR)	24 (19.5–29.5)	28 (22.25–36.75)	25 (20–31)
Age at onset in years, PWE only: median (IQR)	13 (9–15)*	12 (9–15)	13 (9–15)*
Year of onset, PWE only: median (IQR)	2005 (1998–2012)*	2003 (1997–2008)	2005 (1998–2011)*
Type of seizures, PWE only: *n* (%)			
Only nodding seizures	2 (3.3%)	2 (12.5%)	4 (5.2%)
Nodding and other types of seizures	7 (11.5%)	1 (6.2%)	8 (10.4%)
Other types of seizures	52 (85.2%)	13 (81.2%)	65 (84.4%)

*CI*, Confidence interval, *IQR* Interquartile range, *5 data missing, **LOS length of stay, *PWE* Persons with epilepsy

### Prevalence of epilepsy

A total of 78 individuals (62 in Bilomo and 16 in Kelleng) were screened positive using the five questions, giving a suspected epilepsy prevalence of 4.7% in Bilomo and 7.8% in Kelleng. Among them, 70 (89.7%) answered positively to question 1 of the questionnaire, 47 (60.3%) to question 2, 64 (82.1%) to question 3, 20 (25.6%) to question 4 and 75 (96.2%) to question 5.

The 78 suspected cases of epilepsy were examined by the neurologists/physicians with training in epilepsy who confirmed the diagnosis of epilepsy in 77 of them (predictive positive value: 98.7%); the excluded person had experienced only a single seizure by the time of the survey. The crude epilepsy prevalence was therefore 4.6% (61/1321) in Bilomo, and 7.8% (16/204) in Kelleng. Additionally, in 12 PWE (15.6% of participants) head nodding seizures were reported and well described by the families, although no nodding movements were witnessed by the investigators during the study period. Of the 72 PWE who could recall the age of onset of seizures, 70 (97.2%) met the diagnostic criteria for OAE. In Bilomo, 13 (6.7%) households and in Kelleng, one (2.5%) household had two or more PWE, giving an overall proportion of 6.0% of households with multiple cases of epilepsy.

### Comparison between previous and current surveys

The crude prevalence of epilepsy remained stable in Bilomo between 1998 and 2017, 4.9% vs 4.6%, *P* = 0.695 (Table 2). In Kelleng, crude epilepsy prevalence decreased from 10.5 to 7.8% between 2004 and 2018 although this was not statistically significant (*P* = 0.357). However, when standardized for age and sex, the prevalence in Kelleng dropped from 13.5% in 2004 [7] to 9.3% in 2018 (*P* < 0.001).

**Table 2:** Comparison of PWE from the previous and the current surveys in Bilomo and Kelleng

	Bilomo			Kelleng		
	1998	2017	*P*-value	2004	2018	*P*-value
Gender of PWE						
Male: *n* (%)	51 (54.8%)	27 (44.3%)	0.199	10 (52.6%)	8 (50.0%)	0.877
Female: *n* (%)	42 (45.2%)	34 (55.7%)		9 (47.4%)	8 (50.0%)	
Total: *n* (%)	93 (100%)	61 (100%)		19 (100%)	16 (100%)	
Age distribution of PWE: *n* (%)						
0–9 years	3 (3.2%)	1 (1.6%)	< 0.001	0 (0%)	0 (0%)	0.530
10–19 years	56 (60.2%)	12 (19.7%)		6 (31.6%)	2 (12.5%)	
20–29 years	28 (30.1%)	29 (47.5%)		8 (42.1%)	7 (43.8%)	
30–39 years	6 (6.5%)	15 (24.6%)		4 (21.0%)	4 (25.0%)	
40–49 years	0 (0%)	3 (4.9%)		1 (5.3%)	2 (12.5%)	
≥50 years	0 (0%)	1 (1.6%)		0 (0%)	1 (6.2%)	
Total	93 (100%)	61 (100%)		19 (100%)	16 (100%)	
Mean age of PWE ± *SD* years	18.2 ± 5.7	24.9 ± 8.1	*P* < 0.0001	NA	31.4 ± 15.4	NA
Age-specific prevalence: n (%)						
0–9 years	3 (0.5%)	1 (0.2%)	< 0.001	0 (0.0%)	0 (0.0%)	0.676
10–19 years	56 (12.9%)	12 (4.0%)		6 (26.1%)	2 (4.7%)	
20–29 years	28 (11.5%)	29 (17.1%)		8 (30.8%)	7 (30.4%)	
30–39 years	6 (2.6%)	15 (9.4%)		4 (14.8%)	4 (16.7%)	
40–49 years	0 (0.0%)	3 (3.5%)		1 (5.0%)	2 (11.8%)	
≥50 years	0 (0.0%)	1 (0.5%)		0 (0.0%)	1 (2.4%)	
Overall crude prevalence (%)	93/1898 (4.9%)	61/1321 (4.6%)	0.695	19/181 (10.5%)	16/204 (7.8%)	0.357
Age and sex standardized prevalence rate	NA	5.4%	NA	13.5%	9.3%	< 0.001
Age of onset of first seizure: *n* (%)						
Mean age at onset ± *SD* years	12.7 ± 5.97	12.6 ± 4.1	0.909	14.7 ± 6.5	14.1 ± 7.7	0.804
0–2 years	4 (4.3%)	0 (0.0%)	0.944	NA	0 (0.0%)	NA
3–6 years	9 (9.7%)	4 (6.6%)			0 (0.0%)	
7–12 years	33 (35.5%)	23 (37.7%)			8 (50.0%)	
13–20 years	41 (44.1%)	26 (42.6%)			7 (43.8%)	
≥21 years	4 (4.3%)	2 (3.3%)			1 (6.3%)	
Not known	2 (2.2%)	6 (9.8%)			0 (0.0%)	
Total	93 (100.0%)	61 (100.0%)			16 (100.0%)	
Duration of epilepsy: *n* (%)						
Mean duration ± *SD* years	NA	12.7 ± 8.8 yrs	NA	8.7 ± 7.1 yrs	17.4 ± 14.1 yrs	0.036
0–1 year	NA	5 (8.2%)	NA	3 (15.8%)	0 (0.0%)	0.363
2–5 years		10 (16.4%)		4 (21.1%)	1 (6.3%)	
6–10 years		10 (16.4%)		5 (26.3%)	5 (31.3%)	
11–20 years		18 (29.5%)		6 (31.6%)	6 (37.5%)	
> 20 years		13 (21.3%)		1 (5.3%)	4 (25.0%)	
Not known		5 (8.2%)		0 (0.0%)	0 (0.0%)	
Total		61 (100.0%)		19 (100.0%)	16 (100.0%)	

*NA* Not available, *SD* Standard deviation, *PWE* Persons with epilepsy

### Age shift

**Fig. 2:**
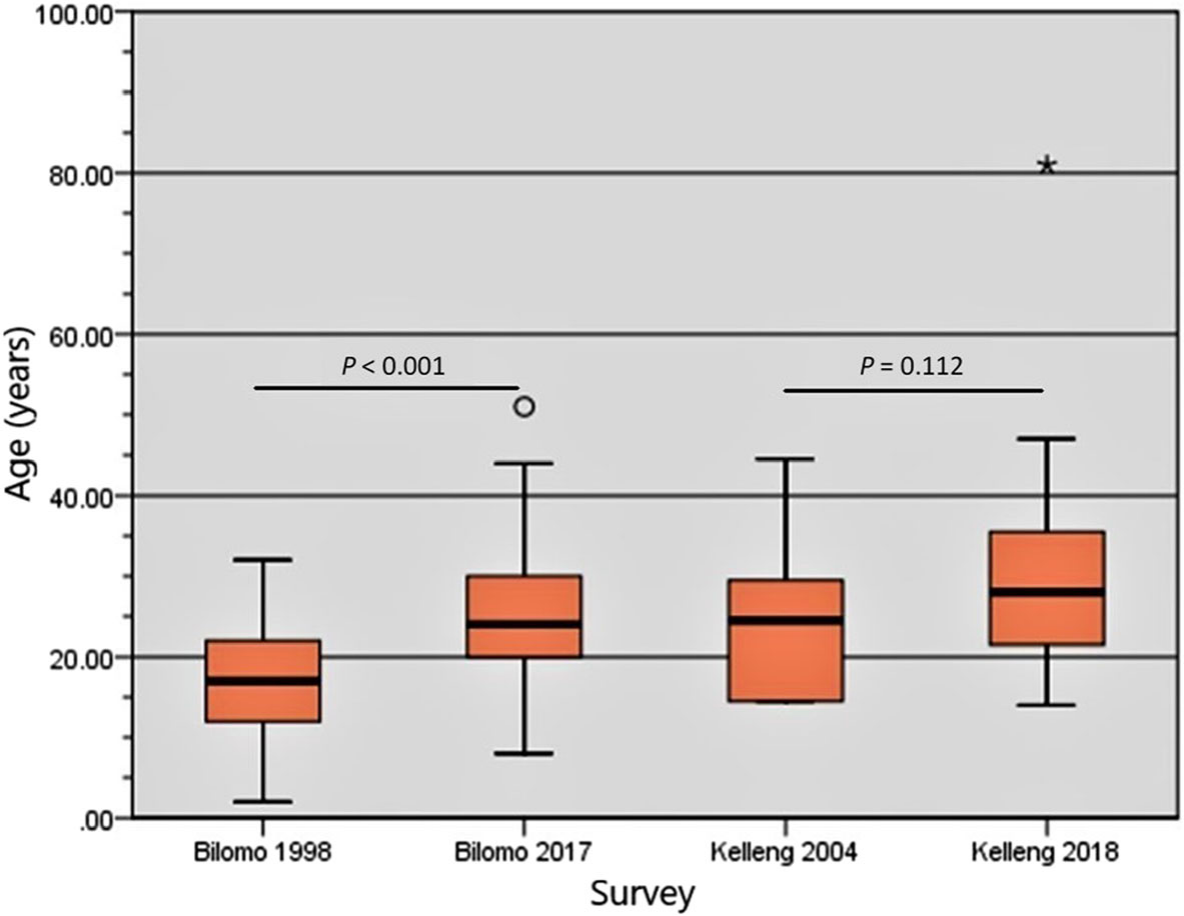
Age shift of cohorts of PWE between 1998 and 2017 in Bilomo and 2004–2018 in Kelleng

**Fig. 3:**
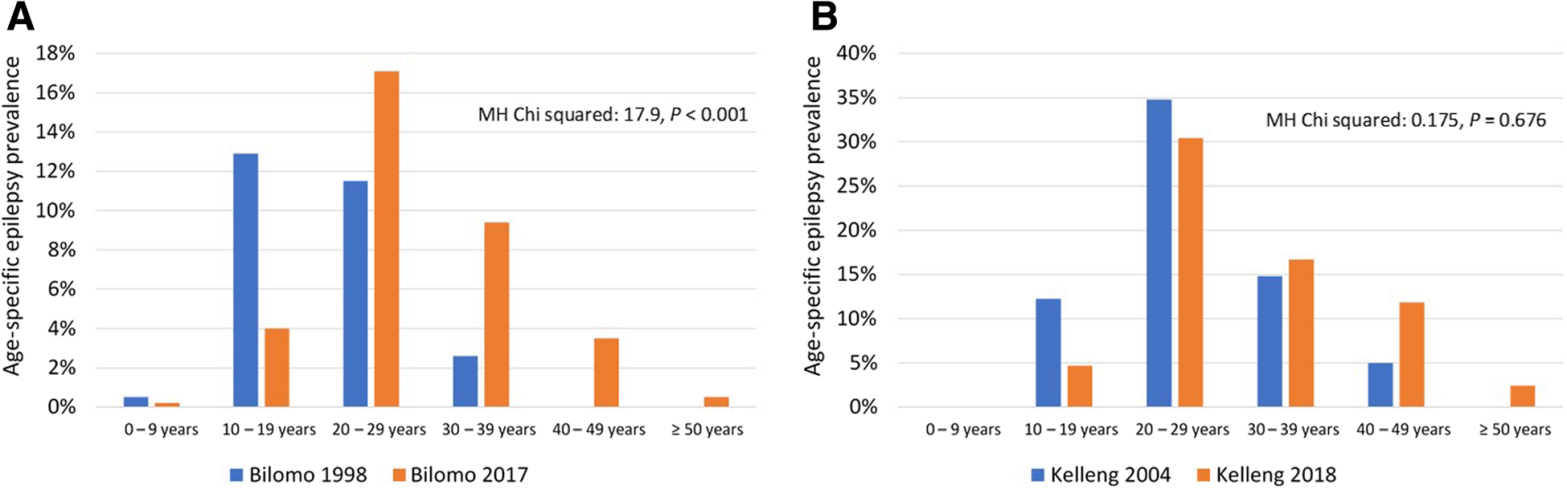
Comparison of age-specific crude prevalence of epilepsy between the previous and the current survey. **a** Bilomo 1998 vs 2017. **b** Kelleng 2004 vs 2018

Considering Bilomo and Kelleng together, the median age of PWE increased from 17 (IQR: 14.5–22) years in 1998/2004 to 25 (IQR: 21–30.5) years in 2017/2018 (*P* < 0.001) (Table 2). At the individual village level, the median age increased from 17 (IQR: 12–22) years to 24 (IQR: 20–30) years in Bilomo (*P* < 0.001); and from 24 (IQR: 14–34) years to 28 (21.25–36.75) years in Kelleng although it was not statistically significant (*P* = 0.112) (Fig. 2). In Bilomo, the 10–19 years age group had the highest epilepsy prevalence (12.9%) in 1998, whereas in 2017 it was the 20–29 years age group, with the prevalence of 17.1% (Fig. 3). The mean age at onset of seizure in Bilomo in 2017 was 12.6 ± 4.1 years and was similar to that recorded in 1998 of 12.7 ± 5.97 years (*P* = 0.909). In Kelleng, the mean age at first seizure was 14.1 ± 7.7 years in 2018 and was comparable to the 14.7 ± 6.5 years recorded in 2004 (*P* = 0.804) (Table 2). In Kelleng, PWE surveyed in 2018 had been living with epilepsy for a longer time (17.4 ± 14.1 years) compared to those surveyed in 2004 (8.7 ± 7.1 years) (*P* = 0.036); data on epilepsy duration was not available for Bilomo in 1998.

### Incidence rates

During the Kelleng 2004 survey, 7 PWE were reported with an onset of epilepsy within the last 5 years for a total survey population of 181 [7]; giving a yearly incidence of 773.5 per 100 000 PY. Meanwhile in 2018, there was only one PWE in Kelleng with an onset of epilepsy within the last 5 years for a total survey population of 204 (Table 2) giving a yearly incidence of 98.0 per 100 000 PY. A drastic decrease in epilepsy incidence (773.5 vs 98.0 per 100.000 PY) was observed in Kelleng between 2004 and 2018 (*P* < 0.001). For the Bilomo 2017 survey, there were 15 PWE with an onset of epilepsy within the last 5 years for a total survey population of 1321 (Table 2), thus a yearly incidence of 227.1 per 100 000 PY.

### Onchocerciasis endemicity and CDTI coverage

**Fig. 4:**
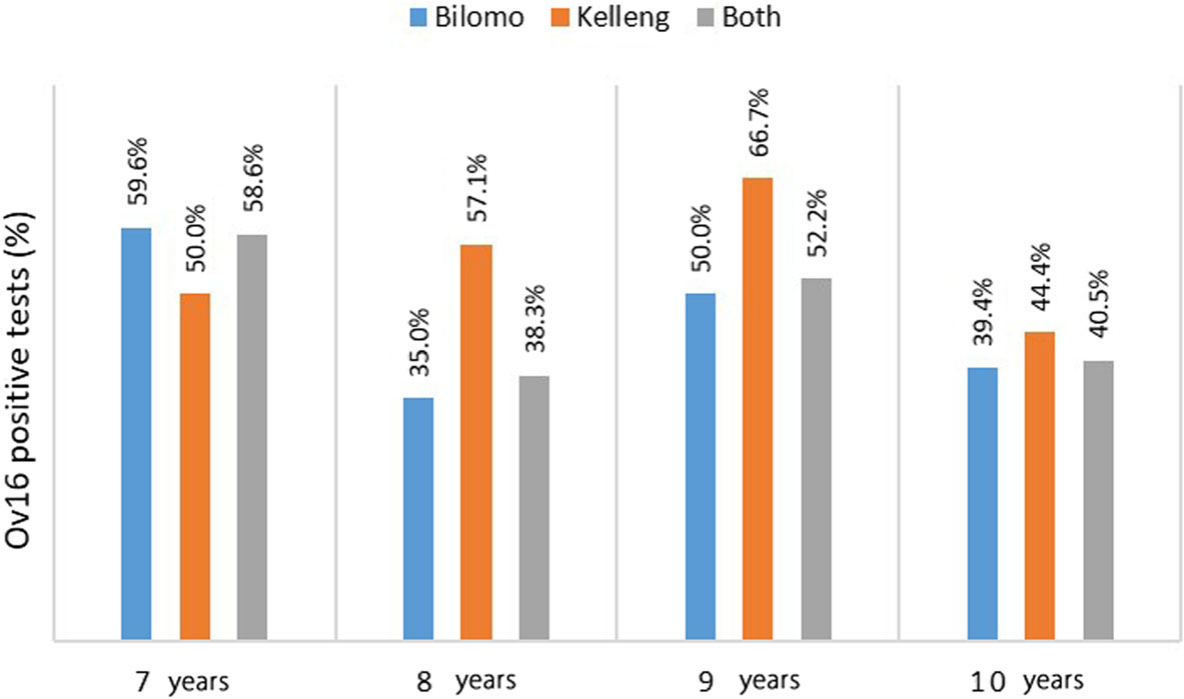
Ov16 results per age group in children aged 7–10 years

Ov16 seropositivity rates amongst children aged 7–10 years was 46.9% (68/145) in Bilomo, and 13/25 (52.0%) in Kelleng; overall Ov16 positivity rate was 47.6%. Age-stratified Ov16 results are shown in Fig. 4. Based on verbal reports of ivermectin use by participants in the households, we estimated CDTI coverage for the year 2017 at 64.1% and 69.2% in Bilomo and Kelleng, respectively.

## Discussion

This study provides additional evidence that CDTI modifies the epidemiology of epilepsy in onchocerciasis-hyperendemic areas. The previous studies (1998 and 2004) as well as current surveys (2017 and 2018) described in this paper, all used a door-to-door strategy and similar instruments for epilepsy screening, thus rendering the results comparable. Our findings suggest that following 19 rounds of annual MDA in Bilomo and 13 rounds in Kelleng, the crude epilepsy prevalence slightly decreased in both villages, more in Kelleng compared to Bilomo, although this change was not significant. However, there was a significant reduction in the standardized epilepsy prevalence in Kelleng between 2004 and 2018. The greater reduction observed in Kelleng could be as a result of an overestimation of the prevalence in 2004; the fact that 16 village residents were not seen during the 2004 survey reduced the denominator and might have inflated the crude prevalence values obtained at the time. If all the 16 absentees did not have epilepsy, the crude prevalence would have been as low as 9.6% as opposed to the reported 10.5%. Both the crude and standardized prevalences reported in our study remained well above the median epilepsy prevalence in Sub-Saharan Africa which is 1.42% [21], in line with previous reports of higher epilepsy prevalence in onchocerciasis-endemic zones [4].

A trend of increased age of PWE compared to previous studies was observed in both villages; one possible explanation is the fact that less children between the ages of 10-20 years developed OAE because annual CDTI decreases the microfilarial load in susceptible individuals every year, thus reducing the risk of attaining a threshold that may trigger epilepsy [3, 9, 22]. We noted that this age shift was statistically significant in Bilomo, but not in Kelleng. The difference is most likely because the initial survey in Bilomo (1998) was conducted in an ivermectin-naïve population unlike Kelleng which had already benefited from at least 10 rounds of ivermectin MDA before the first survey in 2004. The effect of ivermectin in preventing new cases of OAE is further supported by a decreasing epilepsy incidence and significantly higher duration of epilepsy observed in Kelleng 2018 compared to Kelleng 2004, which implies that the same old patients are simply growing older while fewer incident cases of epilepsy occur.

There are several arguments supporting the existence of OAE in these villages; the OAE diagnostic criteria [9] that could be identified in 97.2% of PWE include: the age of onset of epilepsy between 3 and 18 years; a normal psycho-motor development prior to the first seizure; the high epilepsy prevalence in the village with 6% of households having two or more PWE; no obvious cause of epilepsy could be found in the vast majority of cases. It is worth emphasizing that without proper investigations to exclude other causes of epilepsy in an exhaustive manner, this high percentage of suspected OAE cases must be interpreted with caution. Moreover, PWE with nodding seizures, considered as a clinical presentation of OAE [23], were identified: nine in Bilomo and three in Kelleng (Table 1). These findings appear to further strengthen the hypothesis of a causal relationship between onchocerciasis and epilepsy in these areas resulting in a wide spectrum of seizure disorders including nodding seizures. In addition, the occurrence of OAE cases in onchocerciasis-endemic areas requires that a framework for epilepsy management adapted for these settings should be instituted, with a focus on decentralization of care, task shifting to non-physicians and a community-based approach [15, 24, 25].

The results of the Ov16 rapid diagnostic tests were positive for 46.9% of children in Bilomo and 52.0% of those in Kelleng. Such high Ov16 seropositivity rates amongst children below 10 years are overwhelmingly greater than the 0.1% threshold set by the World Health Organization [26] and constitute a major concern, because it indicates a high rate of ongoing onchocerciasis transmission. Additional evidence supporting high transmission rates is the preliminary entomological findings from the Mbam valley in Cameroon, which show that about 98% of infected pools containing blackflies collected between July 2016 and January 2017 were positive for *O. volvulus* [27]. Furthermore, Kamga et al. in 2015 found that communities in the Centre and Littoral Regions of Cameroon were still mesoendemic for onchocerciasis despite 15 years of CDTI [28]. High rates of onchocerciasis transmission create a vicious circle which prolongs the prospects of eliminating the disease because human reservoirs are being multiplied across generations. Ongoing transmission is most probably consequential to the suboptimal ivermectin coverage in both communities; none of the study villages attained 70% coverage in 2017. There is an urgent need for new strategies for onchocerciasis control that will ensure better coverage, minimise microfilarial load, reduce the probability of infective bites by blackflies and ultimately stop onchocerciasis transmission. Various alternative strategies for strengthening onchocerciasis control include addressing perceptions of CDTI coupled with increased community involvement [29], increasing the frequency of ivermectin MDA [30], switching from ivermectin to moxidectin [31, 32] and associating vector control measures to MDA [9, 32].

The positive predictive value for epilepsy diagnosis using at least one positive response to the 5 epilepsy screening questions in our study was 98.7%, more than four times higher than the 22.8% obtained during the validation of the screening tool in 2004 [16]. The difference can be explained by the fact in our study, the screening was done exclusively by physicians as opposed to technicians employed during the validation. Therefore, we observed that the professional background of the interviewer seems to greatly influence the performance of the 5 questions as a screening tool for epilepsy.

The strength of our study is that the baseline and follow-up surveys were performed in both villages with a similar methodology and that documented values for baseline epilepsy prevalence were available. The main limitations of our study include the fact that different teams performed the two surveys in Kelleng (2004 and 2018) and the sample size of the initial epilepsy survey was smaller than the village population, thus leading to a potential overestimation of the crude epilepsy prevalence. In addition, the incidence data we obtained from both villages needs to be interpreted with caution, as this likely represents only estimates of the real situation. Furthermore, for both study sites, years of onset of epilepsy may be influenced by recall bias and this can affect the incidence data. Finally, for standardization purposes, assumptions were made about the stable structure of the population during these years. It is worth mentioning that other risk factors for epilepsy including neurocysticercosis were not systematically excluded. But in a previous case-control study in Bilomo by part of our team, *Taenia solium* infection was not found to be associated with epilepsy [33].

## Conclusions

Despite more than 13 years of CDTI that has reduced the incidence and prevalence of epilepsy, the prevalence of epilepsy in two onchocerciasis-endemic villages of Cameroon remains high, and there is still ongoing transmission of onchocerciasis. The criteria for OAE were met by 97.2% of the PWE with 15.6% presenting with nodding seizures. Our results suggest that onchocerciasis control measures seem to have a significant preventive role in OAE and should be reinforced accordingly.

## Additional files

Additional file 1: Multilingual abstracts in the five official working languages of the United Nations. (PDF 255 kb)

Additional file 2: Method for standardization of population in Bilomo and Kelleng. (XLSX 19 kb)

## Abbreviations

CDTI: Community-directed treatment with ivermectin
IQR: Interquartile range
OAE: Onchocerciasis-associated epilepsy
PWE: Person(s) with epilepsy
RDT: Rapid diagnostic test
