# Supplementary material for: Epidemiology of onchocerciasis-associated epilepsy in the Mbam and Sanaga river valleys of Cameroon: impact of more than 13 years of ivermectin
Source: Infect Dis Poverty. 2018 Dec 3;7:114. doi: 10.1186/s40249-018-0497-1 (PMC6276171; doi:10.1186/s40249-018-0497-1)
Supplement: Supplementary file 1 — Multilingual abstracts in the five official working languages of the United Nations. (PDF 255 kb) [file 40249_2018_497_MOESM1_ESM.pdf]

Translation of the abstract into the five official working languages of the United Nations

الانتشار الوبائي لداء الصرع المصاحب للعمى النهري في أودية أنهار مبام وساناجا في الكاميرون: أثر استخدام عقار ايفرمكتين لأكثر من 13 عاما.

جوزيف نيلسون سيو فودجو، جودوين تاتاه، ارنست نجيه طابة، ليونارد نغاركا، ليونارد نغامنشي نفور، صامويل اريك تشوكوت، ميشال ك. مينجنيجو، فيديل ديما، أوريلين تيلي سيتوك، غريس نكورو، فيليسيان إ. نتون، آن سيسيل تسونج كاني بيسك، سيدريك تشيسنايس، ميشال بوسينيسك، روبرت كليبندرز، ألفريد ك. نجامنشي

مُلَخَّصٌ

الخلفية:لقد تم الإبلاغ عن ارتفاع في انتشار مرض الصرع في العديد من قرى مستوطنة لداء العمى النهري على امتداد أودية أنهار مبام وسانجانا في الكاميرون، بما في ذلك بيلومو وكيلنغ. لقد سعينا إلى تحديد مدى انتشار حالات الصرع بعد أكثر من 13 عامًا من العلاج المجتمعي الموجه بالإيفرمكتين (CDTI).

الطرق: لقد تم إجراء دراسة استقصائية ميدانية في المنازل على كافة السكان المقيمين في القرى في شهر أغسطس 2017 وشهر يناير 2018. وقد تم تشخيص الصرع باستخدام نهج من خطوتين: تنظيم استبيان موحد مكون من 5 بنود يعقبه تأكيد من طبيب أعصاب. وتم استخدام معايير تشخيص كانت قد نُشرت سابقاً لداء الصرع المصاحب للعمى النهري. وتم تطبيق أمصال Ov16 على الأطفال الذين تتراوح أعمارهم من 7 إلى 10 سنوات لتقييم انتقال داء العمى النهري. وقد تم مقارنة النتائج التي تم التوصل إليها مع البيانات السابقة من هتين القريتين.

النتائج: شملت الدراسة الإستقصائية ما مجموعه 1525 فرداً (1321 في بيلومو و204 في كيلنغ) من 233 أسرة في كلتا القريتين.. بلغ المعدل الخام لانتشار مرض الصرع 4.6% في بيلومو (عام 2017) و7.8% في كيلنغ (عام 2018)، بما في ذلك 12 شخصاً مصاب بداء الصرع مع نوبات ارتجاج (15.6% من الحالات). وقد انخفض معدل الإنتشار حسب الجنس والعمر في كيلنغ من 13.5% عام 2004 إلى 9.3% عام 2018.(P < 0.001). وقد تحول متوسط العمر للأشخاص المصابين بداءالصرعمن 17 سنة (IQR: 12–22) إلى 24 سنة (IQR: 20–30) في بيلومو (P < 0.001)، وانخفاضاً ضئيلاً من 24 سنة (IQR: 14–34) إلى 28 سنة (IQR: 21.25–36.75) في كيلنغ (P = 0.112). من جهة أخرى، كان 47.6% من جميع الأطفال الذين تم اختبارهم يملكون مضادات Ov16.

الاستنتاجات: هناك انخفاض في معدل انتشار الصرع في حالات العلاج المجتمعي الموجه بالإيفرمكتين بعد سن13 عام وأكثر في كلتا القريتين. ويشير التحول في الهياكل العمرية إلى أن الإيفرمكتين قد يمنع داء الصرع المصاحب للعمى النهري في إصابة السكان الأصغر سناً. وتشير إيجابية المصل Ov16 إلى استمرار انتقال داء العمى النهري وربما يرجع السبب في ذلك إلى تدابير الوقاية دون المستوى الأمثل. وتثبت النتائج التي توصلنا إليها إلى وجود داء الصرع المصاحب للعمى النهري في الكاميرون، وتؤكد على ضرورة تعزيز برامج مكافحة العمى النهري.

Translated from English version into Arabic by Aya Kurdi and Reham Hussein, through

TRANSLATORS
WITHOUT BORDERS

喀麦隆姆巴姆河谷和萨纳加河谷地区盘尾丝虫病相关癫痫流行病学研究：13 年伊维菌素治疗影响

Joseph Nelson Siewe Fodjo, Godwin Tatah, Earnest Njih Tabah, Leonard Ngarka, Leonard Njamnshi Nfor, Samuel Eric Chokote, Michel K. Mengnjo, Fidèle Dema, Aurélien Tele Sitouok, Grace Nkoro, Félicien E. Ntone, Anne-Cécile Zoung-Kanyi Bissek, Cédric Chesnais, Michel Boussinesq, Robert Colebunders, Alfred K. Njamnshi

摘要

引言：在喀麦隆姆巴姆河谷和萨纳加河谷沿线地区（包括 Bilomo 和 Kelleng）的盘尾丝虫病流行村中，癫痫患病率很高。上述两村已经开展了 13 年以上的面向社区的伊维菌素治疗

（CDTI），本研究旨在评估目前上述两村的癫痫患病率。
方法：2017 年 8 月至 2018 年 1 月,我们在这两个村中对全部常住人口开展了挨家挨户的调查。运用两步法诊断癫痫：首先由被调查人员填写标准化 5 项问卷，然后根据已发表的盘尾丝虫病相关癫痫(OAE)的诊断标准，由神经专科医生确诊。在 7–10 岁儿童中采用 Ov16 血清学检测来评估盘尾丝虫病的传播。最终将本次调查的结果与原来的调查数据进行比较。
结果：两个村落共 233 户 1525 人（Bilomo1321 人和 Kelleng204 人）参与了本次调查。2017 年 Bilomo 的癫痫粗患病率为 4.6%，而 2018 年 Kelleng 的癫痫粗患病率为 7.8%，其中有 12 名癫痫患者伴点头抽搐症状。Kelleng 的年龄和性别标准化患病率从 2014 年的 13.5%下降到 2018 年的 9.3%（$P < 0.001$）。Bilomo 的癫痫患者年龄中位数从 17 岁（IQR: 12–22）上升到 24 岁（IQR: 20–30）（$P < 0.001$），而 Kelleng 仅从 24 岁（IQR: 14–34）上升至 28 岁（IQR: 21.25–36.75）（P = 0.112）。此外，调查发现，在 7–10 岁受测儿童中，47.6%携带 Ov 16 阳性抗体。
结论：Bilomo 和 Kelleng 两个村的居民已经接受了 13 年以上的 CDTI，其癫痫患病率下降。癫痫患者的年龄变化表明伊维菌素或能预防年轻人群罹患 OAE。部分儿童的 Ov16 血清学阳性说明盘尾丝虫病依然在儿童中的传播，可能是因为其防控措施未落实到位。本研究印证了喀麦隆存在 OAE，强调了需要加强盘尾丝虫病的消除工作。

Translated from English version into Chinese by Cong-Shan Liu, edited by Jin Chen

CHINESE CENTER FOR DISEASE CONTROL AND PREVENTION
NATIONAL INSTITUTE OF PARASITIC DISEASES

Epidémiologie de l'épilepsie associée à l'onchocercose (EAO) dans les vallées fluviales de Mbam et de Sanaga au Cameroun : les résultats du traitement à l'ivermectine sur une période de plus de 13 ans

Joseph Nelson Siewe Fodjo, Godwin Tatah, Earnest Njih Tabah, Leonard Ngarka, Leonard Njamnshi Nfor, Samuel Eric Chokote, Michel K. Mengnjo, Fidèle Dema, Aurélien Tele Sitouok, Grace Nkoro, Félicien E. Ntone, Anne-Cécile Zoung-Kanyi Bissek, Cédric Chesnais, Michel Boussinesq, Robert Colebunders, Alfred K. Njamnshi

Résumé
Origine : Un taux élevé de cas d'épilepsie a été signalé dans de nombreux villages endémiques à l'onchocercose le long des vallées fluviales de Mbam et de Sanaga au Cameroun, notamment à Bilomo et à Kelleng. Nous avons cherché à établir la prévalence de l'épilepsie dans ces deux villages après plus de 13 ans de traitement à l'ivermectine sous directives communautaires (TIDC).
Procédés : Des enquêtes porte-à-porte ont été réalisées auprès de l'ensemble de la population dans les villages, en août 2017 ainsi qu'en janvier 2018. Nous avons diagnostiqués les cas d'épilepsie en mettant en oeuvre une stratégie en deux étapes : élaboration d'un questionnaire standard comprenant cinq questions suivi d'une confirmation d'un neurologue. Les critères de diagnostic de l'épilepsie associée à l'onchocercose, antérieurement publiés ont été utilisés. Des tests sérologiques Ov-16 ont été effectués chez des enfants agés entre 7 et 10 ans pour évaluer la transmission de l'onchocercose. Nos résultats ont été comparés à ceux d'études antérieures effectuées dans ces deux villages.
Résultats : 1525 personnes ont été examinées dans les deux villages (1321 à Bilomo et 204 à Kelleng), soit un total de 233 ménages**.** La prévalence brute de l'épilepsie a été de 4,6% dans le village de Bilomo (2017) et de 7,8% dans celui de Kelleng (2018) incluant 12 personnes atteintes d'épilepsie (PWE) (15,6% des cas) accompagnée de troubles de hochement de tête convulsifs. La prévalence normalisée de la maladie selon l'âge et le sexe à Kelleng a diminué de 13.5% en 2004 à

9.3% en 2018 ($P < 0.001$). A Bilomo, l'âge moyen des personnes atteintes d'épilepsie (PWE) a changé allant de 17 (IQR: 12–22) ans à 24 (IQR: 20–30) ans ($P < 0.001$); et à Kelleng, un léger changement est apparu, allant de 24 (IQR: 14–34) ans à 28 (IQR: 21.25–36.75) ans ($P = 0.112$). De plus, 47.6% des enfants examinés et âgés de 7 à 10 ans avaient des anticorps pour lutter contre les antigènes Ov16.
Conclusions : Une diminution de la prévalence de l'épilepsie a été constatée après plus de 13 ans de traitement à l'ivermectine sous directives communautaires (TIDC) dans ces deux villages. Le changement de l'âge des individus observé dans les cas d'épilepsie indique que l'ivermectine peut prévenir l'épilepsie associée à l'onchocercose (EAO) chez les jeunes. La séropositivité révélée par le test OV16 chez les enfants révèle une transmission continue de l'onchocercose, probablement due à des mesures de contrôle sous-optimales. Nos résultats révèlent la présence de l'épilepsie associée à l'onchocercose (EAO) au Cameroun et soulignent la nécessité de renforcer les programmes de lutte et d'élimination de l'onchocercose.

Translated from English version into French by Vero Marie and Akshah Nathan, through

TRANSLATORS
WITHOUT BORDERS

Эпидемиология эпилепсии, связанной с онхоцеркозом, в долинах рек Мбам и Санага (Камерун): эффект применения инвермектина на протяжении более 13 лет

Жозеф Нельсон Сью Фоджо, Годвин Тата, Эрнест Нжи Таба, Леонард Нгарка, Леонард Нжамнши Нфор, Сэмюэль Эрик Шокот, Мишель К. Менгнжо, Фидель Дема, Орельен Теле Ситок, Грейс Нкоро, Фелисьен Э. Нтон, Анн-Сесиль Зунг-Каньи Биссек, Седрик Шесне, Мишель Буссинеск, Роберт Колебундерс, Альфред К. Нжамнши

Аннотация
Общие сведения: Были получены данные о высоких показателях заболеваемости эпилепсией в нескольких эндемичных по онхоцеркозу деревнях, расположенных в долинах рек Мбам и Санага (Камерун), включая деревни Биломо и Келленг. Целью данного исследования было оценить масштабы распространения эпилепсии в этих двух деревнях спустя более 13 лет после начала лечения местного населения инвермектином (ЛМНИ).
Методы: В августе 2017 г. и в январе 2018 г. был проведён поголовный опрос населения указанных деревень. Диагностика эпилепсии проводилась в два этапа: использование стандартного вопросника из пяти пунктов и получение подтверждения невролога. Были использованы ранее опубликованные диагностические критерии эпилепсии, связанной с онхоцеркозом (ЭСО). У детей в возрасте от 7 до 10 лет была взята кровь на серологический анализ на антитела к Ov16 для выявления онхоцеркоза. Результаты были сопоставлены с ранее собранными данными по этим двум деревням.
Результаты: В общей сложности в двух деревнях было опрошено 1525 человек из 233 семей (1321 в Биломо и 204 в Келленге). Общий коэффициент распространенности эпилепсии составил 4,6% в Биломо (2017 г.) и 7,8% в Келленге (2018 г.), включая 12 лиц с эпилепсией (ЛСЭ) (15,6% случаев) с кивательными судорогами. В Келленге стандартизированный по возрасту и полу коэффициент распространенности, в 2004 г. составлявший 13,5%, в 2018 г. снизился до 9,3% ($P < 0{,}001$). Средний возраст ЛСЭ увеличился: в Биломо с 17 лет (диапазон: 12-22 лет) до 24 лет (диапазон: 20-30 лет) ($P < 0{,}001$);

в Келленге с 24 лет (диапазон: 14-34 лет) лет до 28 лет (диапазон: 21,25-36,75 лет) (*P* = 0,112). Кроме того, у 47,6% детей в возрасте с 7 до 10 лет, чья кровь была взята на анализ, были обнаружены антитела к Ov16.
Выводы: Спустя более 13 лет ЛМНИ показатели распространенности эпилепсии в обеих деревнях снизились. Изменения с точки зрения возраста ЛСЭ позволяют предположить, что инвермектин препятствует распространению ЭСО среди молодых людей. Положительная серологическая реакция на Ov16 у детей свидетельствует о том, что заражение онхоцеркозом продолжается; возможно, вследствие недостаточности мер по его предотвращению. На основе полученных данных можно сделать вывод о распространенности ЭСО в Камеруне и о необходимости повышения эффективности программ по борьбе с энхоцеркозом.

Translated from English version into Russian by Dariia and Liudmila Tomanek, through

TRANSLATORS
WITHOUT BORDERS

Epidemiología de la epilepsia asociada a la oncocercosis en los valles de los ríos Mbam y Sanaga de Camerún: el impacto de más de 13 años de invermectina.

Joseph Nelson Siewe Fodjo, Godwin Tatah, Earnest Njih Tabah, Leonard Ngarka, Leonard Njamnshi Nfor, Samuel Eric Chokote, Michel K. Mengnjo, Fidèle Dema, Aurélien Tele Sitouok, Grace Nkoro, Félicien E. Ntone, Anne-Cécile Zoung-Kanyi Bissek, Cédric Chesnais, Michel Boussinesq, Robert Colebunders, Alfred K. Njamnshi

Resumen

Contexto: Se reportó una alta prevalencia de ataques de epilepsia en numerosas poblaciones endémicas de oncocercosis a lo largo de los valles de los ríos Mbam y Sanaga en Camerún, incluídas las poblaciones de Bilomo y de Keling. Buscamos determinar la prevalencia de la epilepsia en estas dos poblaciones en base a más de 13 años de tratamiento con invermectina dirigido por la comunidad (CDTI, por sus siglas en inglés).
Métodos: En agosto de 2017 y enero de 2018, se realizaron encuestas de puerta a puerta al total de los habitantes de estas dos poblaciones. Se diagnosticó la epilepsia utilizando un enfoque de 2 pasos: la distribución de un cuestionario estadarizado de 5 pasos, seguido por la confirmación de un neurólogo. Se utilizó el criterio previamente publicado para diagnosticar la epilepsia asociada a la oncocercosis (OAE, por sus siglas en inglés). Se realizó serología con Ov16 para niños de entre 7 y 10 años para evaluar la transmisión de oncocercosis. Los resultados se compararon con información anterior de estas dos poblaciones.
Resultados: Se encuestó a un total de 1525 individuos (1321 en Bilomo y 204 en Kiling) que habitaban 233 casas**.** La cruda prevalencia de epilepsia fue de 4,6% en Bilomo (2017) y de 7,8% en Keling (2018), incluidas 12 personas con epilepsia (PWE, por sus siglas en inglés) (15,6% de los casos) con convulsiones de cabeza. La prevalencia estandarizada por sexo y edad en Keling decreció de un 13,5%, en 2004, a un 9,3%, en 2018, (*P* <0,001). La edad mediana de PWE cambió de 17 años (IQR: 12-22) a 24 años (IQR: 20-30) en Bilomo (*P* <0,001); y apenas de 24 años (IQR: 14-34) a 28 años (IQR: 21,25-36,75) en Keling (*P* =0,112). Además, el 47,6% de todos los niños testeados de entre 7 y 10 años tenian anticuerpos contra el antígeno Ov16.
Conclusiones: Hay una disminución en la prevalencia de la epilepsia luego de más de 13 años de CDTI en ambas poblaciones. El cambio de edad observado en PWE sugiere que la invermectina

puede prevenir OAE en pobladores jóvenes. La seropositividad al antígeno Ov16 en niños indica que existe transmisión de oncocercosis debido, posiblemente, a medidas de control que se encuentran por debajo de lo óptimo. Nuestros resultados apoyan la existencia de OAE en Camerún y resaltan la necesidad de reforzar los programas de eliminación de la oncocercosis.

Translated from English version into Spanish by PaulaBRod and Felipe Chávez, through

TRANSLATORS
WITHOUT BORDERS
